# Horizontal and Vertical Distributions of Transparent Exopolymer Particles (TEP) in the NW Mediterranean Sea Are Linked to Chlorophyll *a* and O_2_ Variability

**DOI:** 10.3389/fmicb.2016.02159

**Published:** 2017-01-31

**Authors:** Eva Ortega-Retuerta, Maria M. Sala, Encarna Borrull, Mireia Mestre, Fran L. Aparicio, Rachele Gallisai, Carolina Antequera, Cèlia Marrasé, Francesc Peters, Rafel Simó, Josep M. Gasol

**Affiliations:** Biologia Marina i Oceanografia, Consejo Superior de Investigaciones Científicas, Institut de Ciències del MarBarcelona, Spain

**Keywords:** transparent exopolymer particles, chlorophyll *a*, bacteria, carbon, Mediterranean Sea

## Abstract

Transparent Exopolymer Particles (TEP) are relevant in particle and carbon fluxes in the ocean, and have economic impact in the desalination industry affecting reverse osmosis membrane fouling. However, general models of their occurrence and dynamics are not yet possible because of the poorly known co-variations with other physical and biological variables. Here, we describe TEP distributions in the NW Mediterranean Sea during late spring 2012, along perpendicular and parallel transects to the Catalan coast. The stations in the parallel transect were sampled at the surface, while the stations in the perpendicular transect were sampled from the surface to the bathypelagic, including the bottom nepheloid layers. We also followed the short-term TEP dynamics along a 2-day cycle in offshore waters. TEP concentrations in the area ranged from 4.9 to 122.8 and averaged 31.4 ± 12.0 μg XG eq L^−1^. The distribution of TEP measured in transects parallel to the Catalan Coast correlated those of chlorophyll *a* (Chla) in May but not in June, when higher TEP-values with respect to Chla were observed. TEP horizontal variability in epipelagic waters from the coast to the open sea also correlated to that of Chla, O_2_ (that we interpret as a proxy of primary production) and bacterial production (BP). In contrast, the TEP vertical distributions in epipelagic waters were uncoupled from those of Chla, as TEP maxima were located above the deep chlorophyll maxima. The vertical distribution of TEP in the epipelagic zone was correlated with O_2_ and BP, suggesting combined phytoplankton (through primary production) and bacterial (through carbon reprocessing) TEP sources. However, no clear temporal patterns arose during the 2-day cycle. In meso- and bathypelagic waters, where phytoplanktonic sources are minor, TEP concentrations (10.1 ± 4.3 μg XG eq l^−1^) were half those in the epipelagic, but we observed relative TEP increments coinciding with the presence of nepheloid layers. These TEP increases were not paralleled by increases in particulate organic carbon, indicating that TEP are likely to act as aggregating agents of the mostly inorganic particles present in these bottom nepheloid layers.

## Introduction

Transparent Exopolymer Particles (TEP) are defined as a subclass of gel-like organic particles, mainly composed by acidic polysaccharides, that are stainable with Alcian Blue (Alldredge et al., 1993). These particles are widespread in aquatic ecosystems, and their study in the ocean has biogeochemical and applied interests. Due to their high stickiness, TEP act as gluing agents for other particles to form larger aggregates susceptible to sink in the water column, hence stimulating the biological carbon pump (Passow et al., 2001; Burd and Jackson, 2009). However, TEP themselves have low density, and when unballasted, they can ascend through the water column (Azetsu-Scott and Passow, 2004) and accumulate in the sea surface microlayer (Wurl et al., 2011) where they can constitute a major source of primary aerosols (Orellana et al., 2011). The study of TEP has also gained interest in the water desalination industry since they are major agents of reverse osmosis membrane fouling (Berman, 2013). Given the ecological and economic relevance of TEP, there is a need to improve the knowledge about how these substances are distributed in the field and what factors affect their dynamics.

TEP were first observed, and have mostly been described, associated with phytoplankton blooms, in the field (Alldredge et al., 1993; Van Oostende et al., 2012) or in mesocosms and controlled chambers (Engel et al., 2015). From these studies we know that phytoplankton are a major source of TEP and TEP precursors in the sea. However, the relationship between phytoplankton and TEP varies depending on phytoplankton composition and physiology (Passow, 2002; Klein et al., 2011), and environmental variables such as nutrient availability (Mari et al., 2001), turbulence (Pedrotti et al., 2010), or UV irradiation (Ortega-Retuerta et al., 2009a). Therefore, even when phytoplankton are the likely main source for TEP, this is not necessarily translated into predictable relationships in the field between TEP and chlorophyll *a* (Chla), the most used proxy for phytoplankton biomass or production. Elucidating the sources of variability in the TEP-Chla relationships would help predicting the occurrence and dynamics of TEP in the ocean.

In addition to the role of phytoplankton, there are other sources of TEP in the sea, such as macroalgae (Thornton, 2004) or zooplankton (Prieto et al., 2001). Also bacteria are known to modify TEP distributions in the sea in various ways: They colonize and degrade TEP that are released by other organisms (Bar-Zeev et al., 2011; Taylor et al., 2014), thus acting as TEP sinks. Bacteria can also directly release TEP (Ortega-Retuerta et al., 2010) so they constitute TEP sources themselves. Finally, bacterial interactions with phytoplankton mediate TEP release (Van Oostende et al., 2013) and induce changes on their formation rates and properties such as their stickiness (Rochelle-Newall et al., 2010). The relative importance of these mechanisms governing the TEP dynamics in aquatic habitats, specifically in the Mediterranean Sea, remains unexplored.

The published information on TEP distributions in the Mediterranean Sea is particularly scarce (Prieto et al., 2006; Ortega-Retuerta et al., 2010; Bar-Zeev et al., 2011). The few published studies, however, concur in that TEP stocks are high when compared to other oceans. For instance, maximum TEP concentrations (up to 11,000 μg Xeq. L^−1^ in surface waters) were observed in Adriatic Sea samples (Passow, 2002). Exceptionally high in the Mediterranean Sea are the relative TEP concentrations with respect to Chla concentrations; higher TEP/Chla ratios than in other ocean basins have been taken to suggest that TEP are an important fraction of the particulate organic matter pool, and likely important drivers of carbon and particle fluxes in this oligotrophic sea.

Here, we report for the first time TEP distributions in the Catalan Sea (NW Mediterranean). Our specific goals were: (1) to determine the potential drivers of TEP from a wide range of physicochemical and biological variables and (2) to examine the variability in the TEP-Chla relationship across multiple spatial and temporal scales.

## Materials and methods

### Study site and sampling

**Figure 1:**
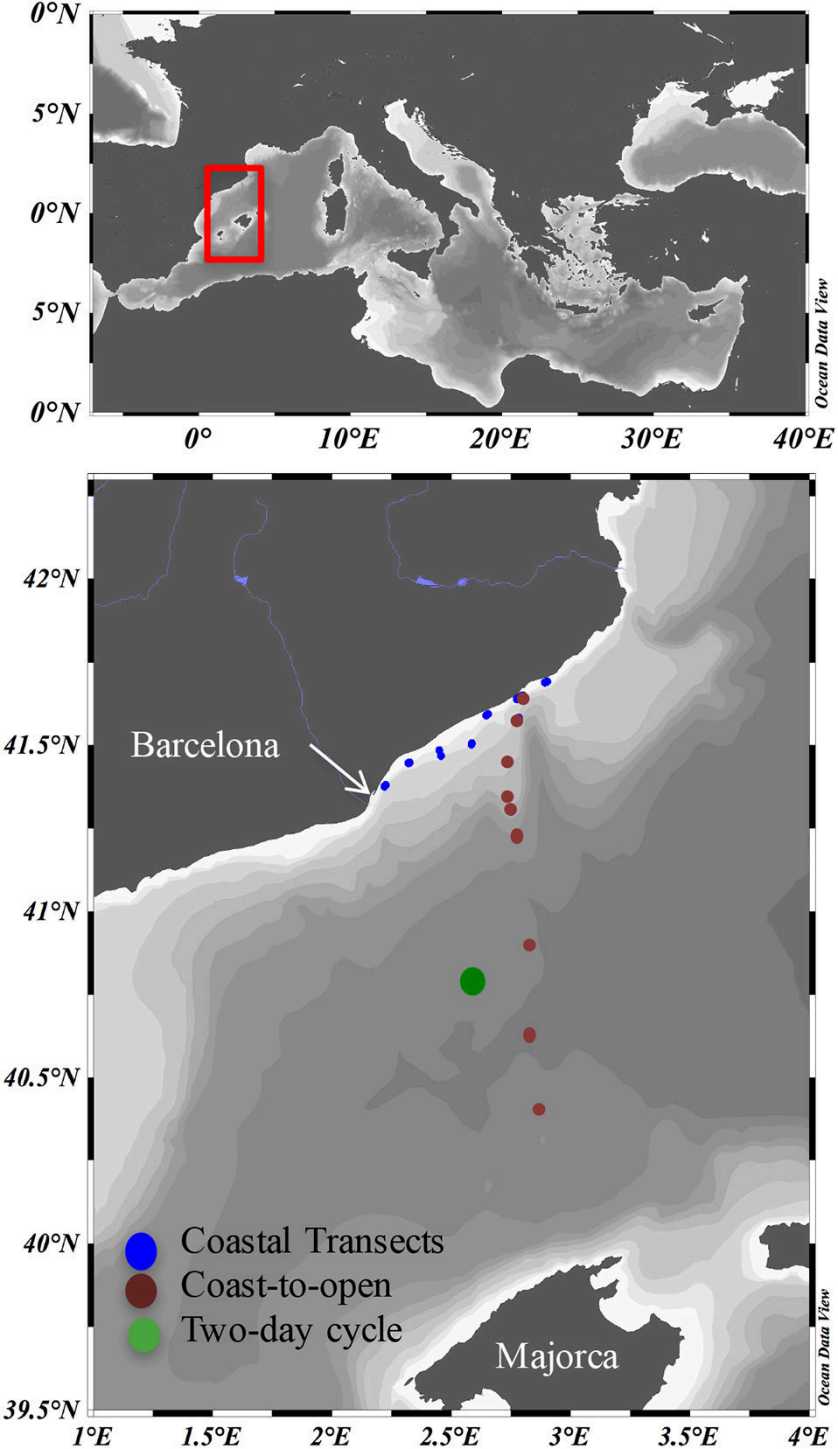
**Study area**. Blue symbols, Coastal transects. symbols, Coast-to-open sea transect. Green symbol, 2-day cycle.

Samples were taken during the cruises NEMO1, NEMO2, and SUMMER2 in Mediterranean waters between the Catalan Coast and north of Majorca Island on board the Spanish RV “García del Cid” (Figure 1). Transects parallel to the Catalan coast (following the bathymetry line at 40 m bottom depth) between Barcelona and Blanes were conducted in May 10th (transect 1) and June 11th (transect 2, Figure 1). During these transects, surface (2 m) samples were taken every hour from the underway continuous flow with the ship moving at ~7 knots, so that each sample was taken approximately at every 12 km. A coast-to-offshore transect was performed during NEMO1, from May 11th to 20th, including stations located in the shelf (Stations 1 and 2), slope (stations 3, 4, and 5) and basin (stations 6, 8, and 9). Station 7 was sampled during NEMO2, 1 month later (June 12th). Water samples in these transects were collected using a rosette (12 Niskin bottles with external spring, 12 L each) coupled to a Sea-Bird Conductivity-Temperature-Depth profiler, a WET Labs C-Star transmissometer and a SeaPoint optical backscatter sensor. Up to six depths were sampled from each station, from surface to bottom (down to 2300 m) waters including the surface, the O_2_ maximum, the deep chlorophyll maximum (DCM) when present, mesopelagic waters, and bottom nepheloid layers.

The 2-day lagrangian study (SUMMER2 cruise) was conducted aboard R/V “García del Cid” from 22nd to 24th May at ca. 45 nautical miles from the coast, within the core of a cyclonic eddy over a water-column depth of ca. 2000 m. A Lagrangian drifter was deployed to track the movement of the upper 15-m water layer. Each drifter consisted of a spherical floatable enclosure that contained a GPS and an emitter, from which 10 m cylindrical drogues hanged 5 m below the sphere. The drifters sent their position every 30 min, and all ship operations were conducted next to them. Samples were taken with the rosette every 4 h at six depths from surface to 200 m.

### Chemical and biological analyses

TEP were analyzed following the colorimetric method proposed by Passow and Alldredge (1995). Samples (250–500 mL) were filtered through 25 mm diameter 0.4 μm pore size Polycarbonate filters (DHI) at low pressure (100 mm Hg). The filters were stained with 500 μL of Alcian Blue (0.02%, pH 2.5) for 5 s and rinsed with MilliQ water. The filters were soaked in 80% sulfuric acid for 3 h and the absorbance of the extract was determined at 787 nm in a Varian Cary spectrophotometer. Duplicates were taken for each sample. Previous analyses have shown a CV of 13% between TEP replicated measurements with this method (details not shown). We have calculated an average range of ±30.9% between duplicates in our dataset. Duplicate blanks (empty filters stained with alcian blue) were also taken at every station. The Alcian Blue dye solution was calibrated just before the cruise using a standard solution of xanthan gum processed with a tissue grinder and subsequently filtered through two sets of filters (five points in triplicate).

Chla concentration was determined by filtering 150 mL of seawater on GF/F filters (Whatman), extracting the pigment in acetone (90% v:v) in the dark at 4°C for 24 h, and measuring fluorescence with a Turner Designs fluorometer.

Analyses of dissolved inorganic nutrient concentrations [nitrate (NO_3_), nitrite (NO_2_), phosphate (PO_4_), and silicate (SiO_2_)], were done by standard segmented flow analyses with colorimetric detection (Hansen and Grasshoff, 1983) using an Seal Analytical AA3 High Resolution AutoAnalyzer.

Particulate organic carbon (POC) was measured by filtering 1000 mL of seawater on pre-combusted GF/F glass fiber filters (4 h, 450°C). The filters were frozen in liquid nitrogen and kept at −80°C until analysis. Prior to analysis, the filters were dried at 60°C for 24 h. Then the filters were dried again and analyzed with a C:H:N autoanalyser (Perkin-Elmer 240).

For bacterial abundance samples, 1.8 ml were preserved with 1% paraformaldehyde + 0.05% glutaraldehyde (final conc.) and frozen in liquid nitrogen until processed in the lab. Bacterial abundance (BA) was analyzed by flow cytometry (FACSCalibur cytometer, Becton and Dickinson) after staining with SYBRGreen I (Molecular probes). Bacteria were detected by their signature in a plot of side scatter vs. FL1 (green fluorescence) as explained in Gasol and del Giorgio (2000).

Bacterial Production (BP) was estimated using the ^3^H-leucine incorporation method described by Kirchman et al. (1985). Three 1.2-mL aliquots and two trichloroacetic acid (TCA)-killed controls (5% final concentration) of each sample were incubated with 40 nmol L^−1, 3^H-leucine (epipelagic samples) or 80 nmol L^−1, 3^H-leucine (meso- and bathy-pelagic samples). The incubations were carried out in a water bath at *in situ* temperature in the dark. The incorporation was stopped by adding cold TCA (5% final concentration) to the vials, and samples were kept at −20°C until processing as described by Smith and Azam (1992). Radioactivity was then counted on a Beckman scintillation counter. Leucine incorporation rates were converted into carbon production using the conversion factor of 1.55 kg C produced per mole of leucine incorporated and considering no isotope dilution (Simon and Azam, 1989).

Given that TEP are frequently enriched in fucose (Zhou et al., 1998), we determined fucosidase activity using a fluorogenic substrate, as in Sala et al. (2016). Each sample (350 μl) was pipetted in quadriplicates into 96 black well-plates, with 50 μl of the substrate 4-methylumbelliferyl β-D-fucoside (Sigma-Aldrich) at a final concentration of 125 μM. Fluorescence was measured immediately after addition of the substrate and after incubations, at *in situ* temperature and in the dark, for 15, 30 min, 1, 3, and 5 h. The measurements were done with a Modulus Microplate (DISMED, Turner BioSystems) at 450 nm excitation and 365 nm emission wavelengths. The increase of fluorescence units during the period of incubation was converted into enzymatic activity with a standard curve prepared with 4-methylumbelliferone (MUF, Sigma-Aldrich).

### Statistical analyses

We used the Statistica 7.0 software package to test the potential drivers of TEP distributions across the different spatial and timescales. We performed pairwise Pearson correlations between TEP concentrations and the following physico-chemical and biological variables: Temperature, salinity, turbidity, O_2_, nutrients (NO_3_, PO_4_, SiO_4_), particulate organic carbon (POC) and nitrogen (PON), chlorophyll *a* (Chla), bacterial abundance (BA), bacterial production (BP), and extracellular fucosidase activity. Data were log_10_-transformed and Bonferroni-corrected when needed.

## Results

### Horizontal TEP distribution along the catalan coast

**Figure 2:**
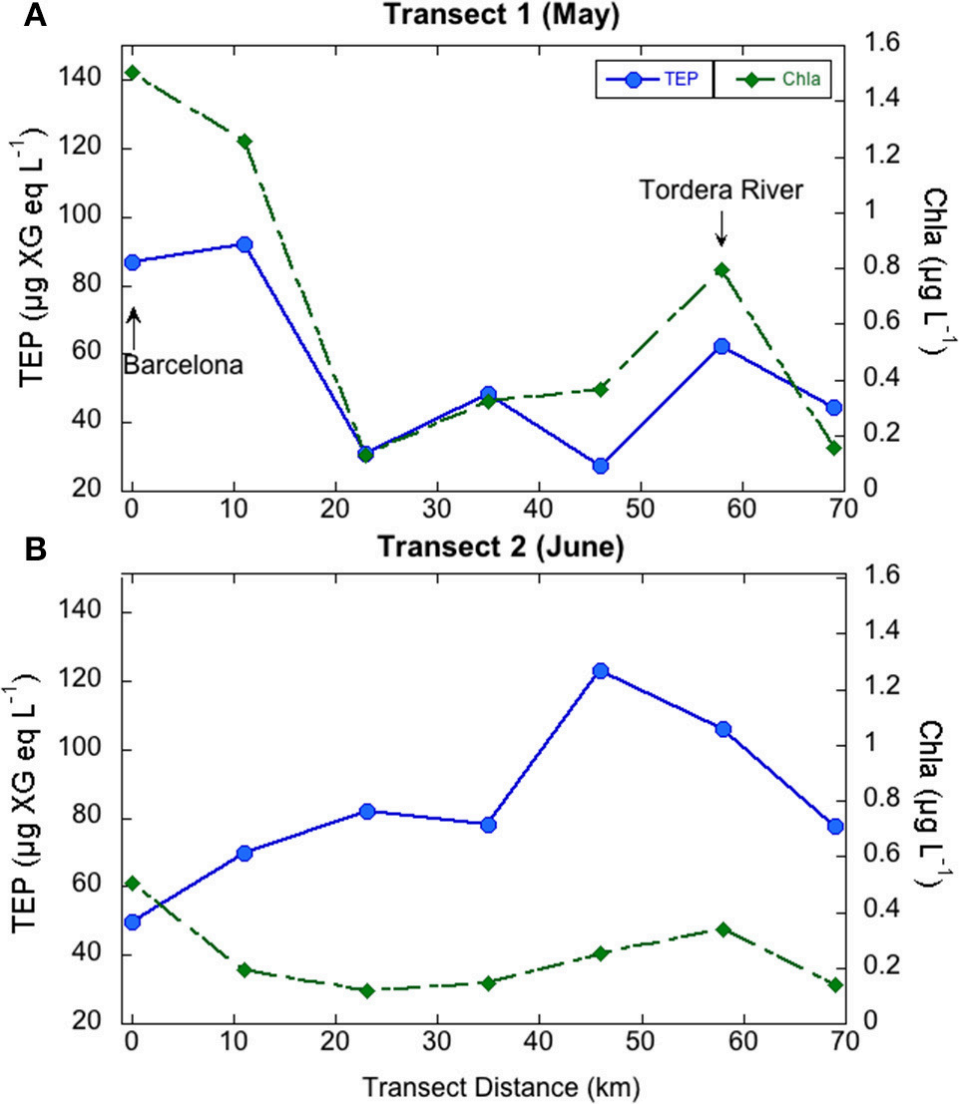
**Variations of TEP (blue symbols) and chlorophyll ***a*** (green symbols) concentration in the coastal transects performed in May (A)** and June **(B)**.

**Figure 3:**
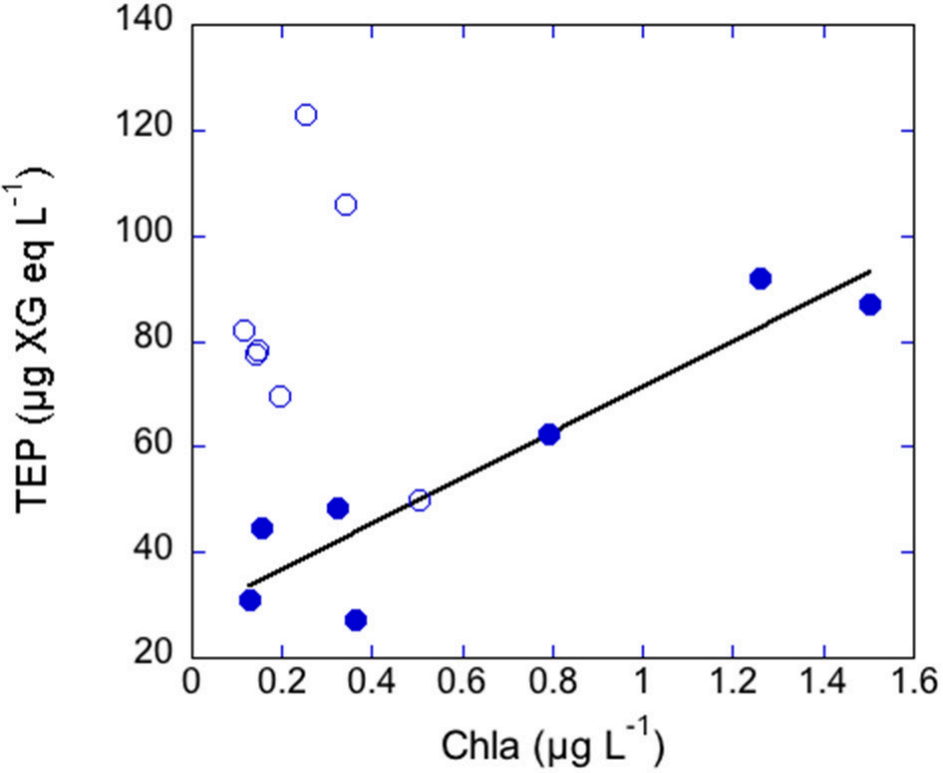
**Scatterplot between Chla and TEP concentrations in the coastal transects performed in May (filled symbols) and June (open symbols)**. TEP were related to Chla in May (*r* = 0.93, *p* < 0.001, *n* = 7).

In the coastal transects, TEP concentrations ranged from 27.4 to 122.8 μg XG eq L^−1^, and were overall higher in June (average 83.7 ± 23.9 μg XG eq L^−1^) than in May (average 56.1 ± 25.8 μg XG eq L^−1^; Table 1). In contrast, Chla concentrations were overall higher in May (0.65 ± 0.55 μg L^−1^) than in June (0.24 ± 0.14 μg L^−1^) and BP rates were similar in the two transects (0.18 ± 0.16 μg C L^−1^ h^−1^, ranging from 0.91 to 10.97, in May, 0.14 ± 0.21 μg C L^−1^ h^−1^, ranging from 0.50 to 14.79 in June). Dissolved inorganic nitrogen (DIN, nitrate+nitrite+ammonia) averaged 0.65 μM in May, ranging from 0.19 to 1.28 μM, and averaged 0.41 μM in June, ranging from 0.21 to 1.02 μM. Dissolved phosphate concentrations averaged 0.07 μM in May, ranging from 0.05 to 0.10 μM, and averaged 0.06 μM in June, ranging from 0.05 to 0.10 μM (Supplementary Figure 2). In May, TEP showed maxima in waters near Barcelona and north of the outflow of the Tordera River (Figure 2A). In these locations DIN concentration was 1.2 μM and phosphate concentration was 0.093 μM, two to eight-fold higher than in the rest of the stations. TEP-values were significantly correlated to Chla concentration (*r* = 0.93, *p* = 0.0003, *n* = 7) and marginally correlated to BP (*r* = 0.72, *p* = 0.06, *n* = 7, Figure 3). In June, TEP distributions showed maxima south of the outflow of the Tordera River, and were uncorrelated to Chla (Figure 2B) nor to BP. The TEP/Chla ratios were markedly higher in June (434.0 ± 197.5) than in May (136.7 ± 91.0, Table 1).

**Table 1:** **Ranges of TEP concentration and TEP/Chla ratios in the different transects, depth profiles and diel cycles presented here**.

	**TEP (μg XG eq L^−1^)**	**TEP/Chla**	***n***
Coastal transect May	27.4–92.1	57.7–283.4	7
Coastal transect June	49.8–122.8	98.4–706.7	7
Epipelagic	4.9–54.2	18.1–316.8	36
Meso- and bathy-pelagic	5.2–19.0	–	23
2-day cycle	5.7–55.9	15.3–1217.6	78

*n, number of samples*.

### Horizontal TEP distribution from coastal to open sea waters

The concentration of TEP in the studied coast-to-open sea transect ranged from 4.9 to 54.2 μg XG eq L^−1^ with a mean concentration of 18.7 ± 11.4 μg XG eq L^−1^.

**Figure 4:**
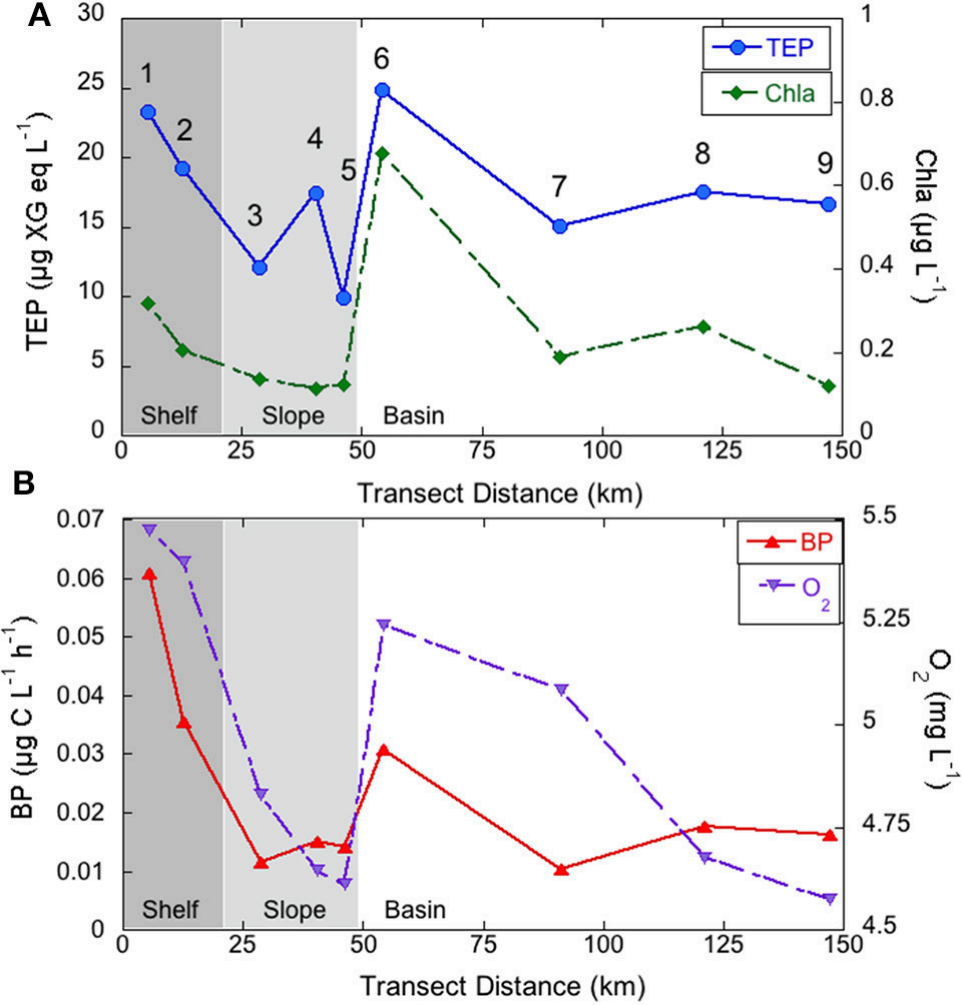
**Depth-averaged TEP concentration (μg XG eq L^**−1**^) and Chla (μg L^**−1**^) (A)** and BP (μg C L^−1^ h^−1^) and O_2_ (mg L^−1^) **(B)** in epipelagic waters of the coast to open sea transect.

We calculated depth-averaged TEP concentrations in epipelagic waters (0–200 m) in an attempt to look at horizontal distribution patterns. Depth-averaged epipelagic TEP ranged from 9.9 to 24.9 μg XG eq L^−1^ (Figure 4A). TEP concentrations were highest near the coast and 60 km offshore, at the slope to basin transition (station 6, Figure 4A). The same horizontal patterns were observed for Chla, O_2_, BP (Figure 4B), rendering significant correlations with TEP (*r* = 0.7, *p* < 0.05, *n* = 9). TEP were likewise related to POC (*r* = 0.9, *p* < 0.01, *n* = 9) and to the ratio between BP and O_2_(*r* = 0.7, *p* < 0.05, *n* = 9), which can be considered a proxy of bacterial reprocessing of photosynthetically fixed carbon.

### TEP vertical distribution in epipelagic waters

**Figure 5:**
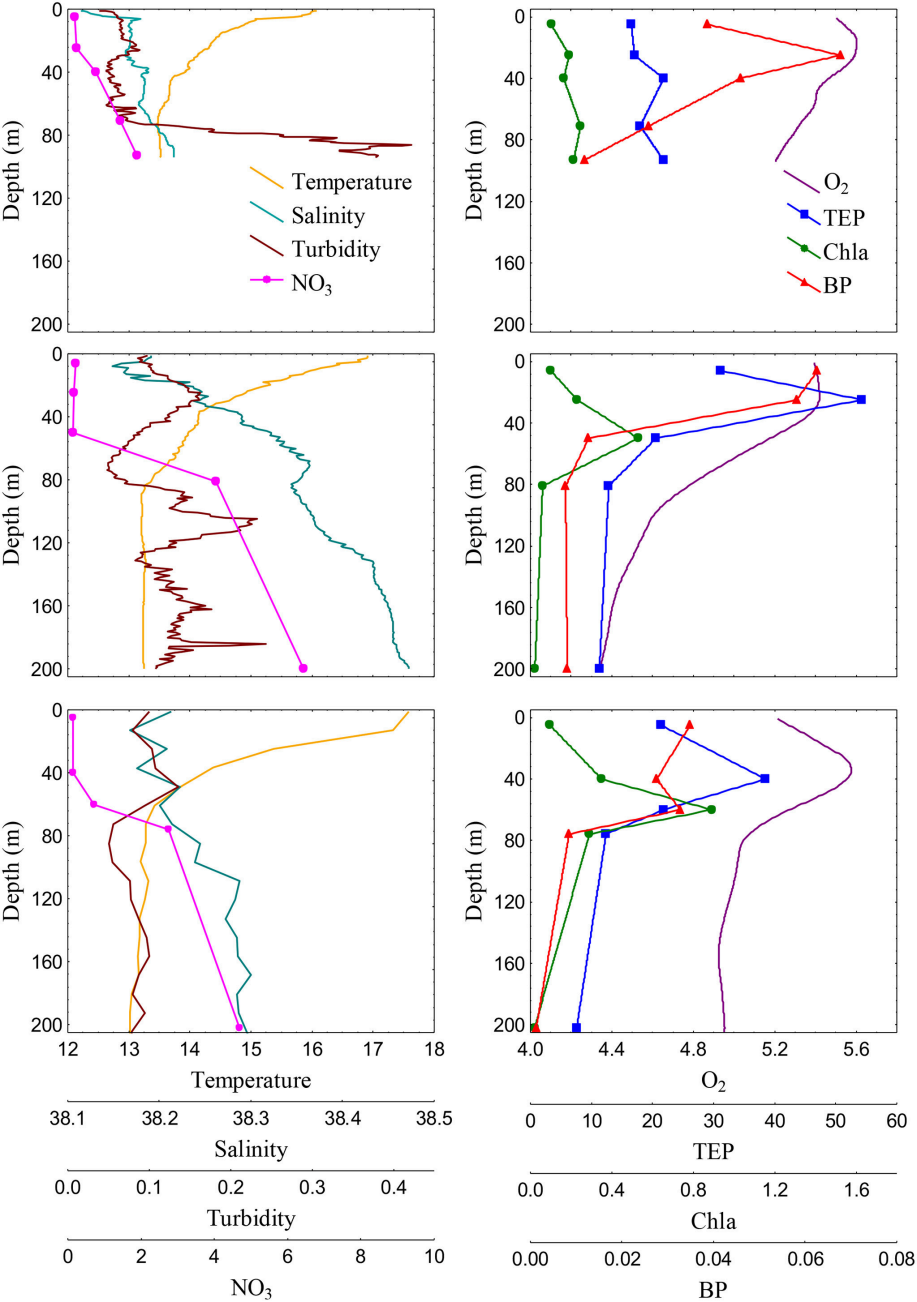
**Vertical profiles of Temperature (°C), salinity (practical salinity units), turbidity (FTU) and NO_**3**_ (μM) (left panels) and O_**2**_ (mg L^**−1**^), TEP concentration (μg XG eq L^**−1**^), Chla (μg L^**−1**^) and BP (μg C L^**−1**^ h^**−1**^) (right panels) in epipelagic waters of station 2 (shelf, upper panels), station 4 (slope, middle panels), and station 8 (basin, bottom panels)**.

TEP vertical distribution patterns were variable among stations. Shelf waters exhibited mixed temperature and salinity profiles (Figure 5) and other chemical and biological variables were also quite uniform in the vertical profile. In these stations, Chla ranged from 0.10 to 0.38 μg L^−1^, BP ranged from 0.012 to 0.078 μg C L^−1^ h^−1^, and POC ranged from 3.7 to 7.2 μM. TEP vertical distributions were also quite homogenous from surface to the bottom, averaging 24.9 ± 3.0 and 19.7 ± 2.5 μg XG eq L^−1^ in stations 1 and 2, respectively (Figure 5). Conversely, well-developed DCM were detected in slope and basin waters between 50 and 60 m, with Chla concentrations ranging from 0.41 (station 5, slope) to 1.73 (station 6, basin) μg L^−1^. The TEP/Chla ratios ranged from 16.8 (station 6, DCM) to 316.0 (station 3, surface). They were generally higher at the surface and lower at the DCM. Bacterial production ranged from 0.005 to 0.065 μg C L^−1^ h^−1^ and the vertical distribution varied between stations: at the slope stations, BP was highest at the surface and subsurface, while in basin stations BP showed a bimodal profile, with peaks at the surface and at the DCM. POC concentrations, that ranged from 2.9 to 11.5 μM, showed similar distributions than TEP in the slope stations (with surface or subsurface peaks) but covaried with Chla, with maxima at the DCM, in the basin stations. TEP also showed marked vertical changes in slope and basin waters (Figure 5): they generally peaked at the surface (slope stations 3 and 5) or at the subsurface (slope station 4 and basin stations). These subsurface maxima were located between 25 and 55 m and showed values from 33.0 to 54.2 μg XG eq L^−1^ (mean concentration 39.6 ± 10.3 μg XG eq L^−1^). TEP maxima were always located shallower than the DCM and coincided with O_2_ maxima, and nutrient minima (Figure 5). At the slope, TEP maxima were also coincident with BP and POC maxima, while the Chla maximum was always deeper. By contrast, in the basin, BP and POC coincided with Chla while TEP and O_2_ peaked at shallower depths.

### Drivers of TEP vertical distribution

We assessed which were the environmental drivers of TEP vertical distributions in epipelagic waters performing Pearson correlation tests between TEP concentrations and a number of physical (temperature, salinity, turbulence), chemical (nutrients, O_2_), and biological (Chla, BP, bacterial abundance, bacterial fucosidase activity) variables (Table 2). Since vertical distribution patterns of TEP and biological variables such as Chla and BP differed among shelf, slope and basin stations, we separated shelf stations from the others for the analysis. TEP was never significantly correlated to Chla. In shelf waters, TEP was not related to any other physicochemical or biological parameter. In slope and basin waters, TEP was significantly and positively correlated to O_2_, BP and the ratio BP/O_2_, and significantly negatively correlated to nutrients and N/P ratios (Table 2).

**Table 2:** **Results of Pearson correlations between TEP concentration and different physical, chemical, and biological variables measured in epipelagic waters during the NEMO cruises at the slope and basin stations**.

**Dependent variable**	**Independent variable**	***r***	***p***
**SLOPE AND BASIN STATIONS (*n* = 25)**			
TEP	Temperature	0.55	0.004
	Turbidity	0.68	0.000
	O_2_	0.83	0.000
	NO_3_	−0.82	0.000
	PO_4_	−0.58	0.005
	N/P	−0.56	0.008
	SiO_4_	−0.73	0.003
	POC	0.79	0.000
	Chla	ns	
	Bact. Ab.	ns	
	Bact. Prod.	0.70	0.000
	Bact. Fuc.	ns	
	BP/O_2_	0.68	0.000

*r, correlation coefficient; p, level of significance*.

### TEP vertical distribution in mesopelagic and bathypelagic waters

TEP concentrations were half lower in meso- and bathypelagic waters with respect to the epipelagic, showing minima between 4.9 and 11.2 μg XG eq L^−1^. By contrast, relative TEP increases were observed in waters near the bottom in all slope and basin stations, parallel to increases in turbidity (proxy of total particle concentration).

TEP in meso- and bathypelagic waters were only significantly related to turbidity (*r* = 0.54, *p* < 0.01 *n* = 23), indicative of the relevance of bottom nepheloid layers (BNL). Remarkably, TEP were uncorrelated with POC in these layers. No significant correlation was found between TEP and BP, but a correlation with bacterial fucosidase activity (*r* = 0.45, *p* < 0.05 *n* = 23) could be observed.

### Diel TEP variations

**Figure 6:**
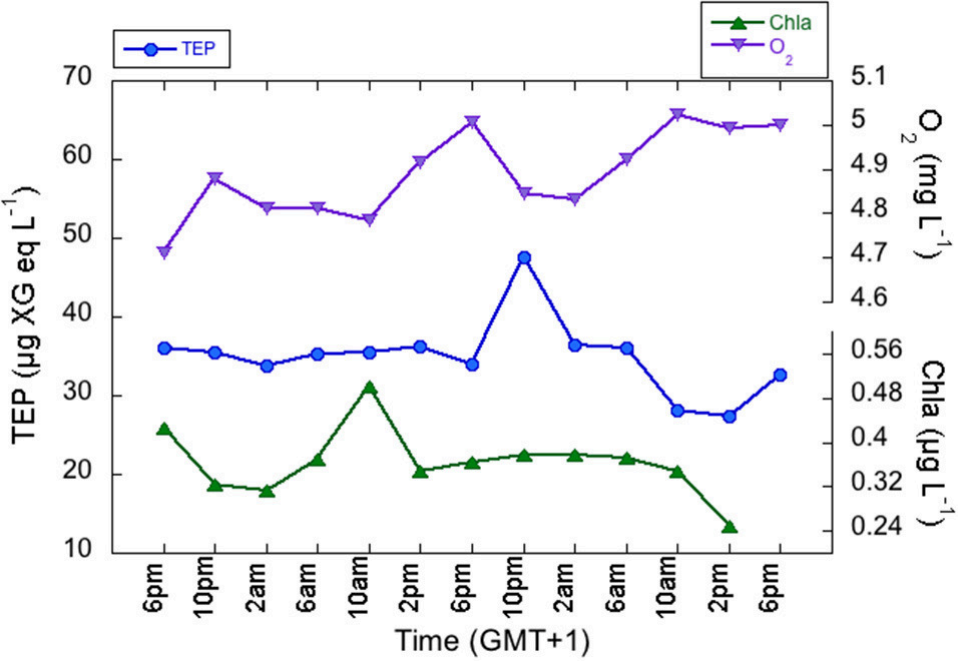
**Diel variations of depth-averaged TEP (blue circles), Chlorophyll a (green triangles), O_**2**_ (purple triangles) concentration**.

The same surface water mass was sampled over time during the 2-day lagrangian study in the SUMMER2 cruise, as confirmed by plotting depth-averaged temperature and salinity values of each CTD cast (Supplementary Figure 1), except for the last two casts were a warmer water mass was likely sampled. We did not detect a recurrent diel pattern of TEP or of any other biological variables such as Chla or BP (Figure 6), even though these variables varied highly during the cycle. Chla showed subsurface maxima of 0.75–1.33 μg L^−1^ between 40 and 54 m. TEP concentrations ranged ten-fold, from 5.7 to 55.9 μg XG eq L^−1^ (average 34.1 ± 5.7 μg XG eq L^−1^). TEP maxima were situated between 25 and 47 m with highest values during the night (10 p.m. and 2 a.m.). These maxima were always shallower than the DCM, and were again vertically coincident with O_2_ and BP maxima. TEP concentrations were not significantly correlated to Chla, but a significant correlation was observed with O_2_ (*r* = 0.70, *p* = 0.000, *n* = 78), BP (*r* = 0.48 *p* = 0.000, *n* = 78), and the ratio BP/O_2_ (*r* = 0.44, *p* = 0.0001, *n* = 78). We looked at temporal variations of TEP, Chla, O_2_, and BP using depth-averaged values for all the epipelagic depths. Diel dynamics of TEP, Chla, O_2_, and BP were not coupled, but we detected Chla and O_2_ increases that were followed by a TEP increase after 4–12 h (Figure 6). However, lagged correlations between these variables, using either maxima or integrated values, were not significant.

## Discussion

TEP dynamics can affect particle aggregation rates because of stickiness, and particle sinking rates due to their low density. Thus, determining and predicting TEP dynamics is crucial if we want to accurately estimate carbon and particle fluxes. The TEP-values observed in this study are within the range of data published for Mediterranean Sea waters (Prieto et al., 2006; Ortega-Retuerta et al., 2010; Bar-Zeev et al., 2011) and at the lower range of other coastal ocean areas (Klein et al., 2011; Van Oostende et al., 2012). By contrast, the TEP/Chla ratios were in the upper range of those previously published for other ocean basins (Prieto et al., 2006), except for those measured in the Mediterranean Sea (Ortega-Retuerta et al., 2010; Bar-Zeev et al., 2011).

### TEP variation in the coastal transects

TEP maxima were observed near the city of Barcelona and near the outflow of the Tordera River, areas with higher nutrient concentrations. Coincidentally, there are two desalination plants located at the mouth of the Llobregat (next to Barcelona) and Tordera Rivers, whose feedwater intake pipes are located between 800 and 2200 m from the coast and at 30 m depth, although they are not always operative. Given that TEP are directly linked to membrane fouling, it is important to be able to predict TEP occurrence in these locations. In May, TEP variations in these coastal waters could be explained by variations in Chla, suggesting a direct linkage between phytoplankton and TEP at this geographical scale. However, this relationship was absent in June (Figure 2). The higher TEP/Chla ratios in June were due to both higher TEP concentrations than in May and lower Chla concentrations than in May. Indeed, primary productivity (PP) is on average two-fold higher in May than in June in coastal NW Mediterranean Sea waters (Gasol et al., 2016). Bacteria did not seem to be a significant TEP source in June as no significant correlations between TEP and BP or TEP and the BP/Chla ratios were observed. Hence, TEP may accumulate in the sea surface at the beginning of the stratification period, similar to other phytoplankton-derived organic matter (Avril, 2002; Vila-Reixach et al., 2012; Romera-Castillo et al., 2013). These substances may be not taken up immediately by bacterioplankton due to nutrient defficiency, which is common in summer (Sala et al., 2002; Pinhassi et al., 2006). What we could resolve in this analysis was that the distribution of TEP in coastal waters could be predicted from Chla, a variable that is frequently monitored, but only at certain periods of the year.

### TEP horizontal distribution from coastal to offshore waters

Our dataset also allowed concluding that Chla is a good predictor of TEP concentrations at the horizontal scale from the coast to the open sea, since significant positive relationships were observed between these two variables. This information is important because it would allow estimating TEP concentrations, for instance, using remote sensing Chla values. It is worthy to mention that, if we restrict the analysis to surface values only instead of depth-averaged values, TEP and Chla were also related (*r* = 0.63 *p* = 0.06, *n* = 9), while not remarkable covariations were found between TEP and other biological variables. This reinforces the possibility of using remote sensing Chla data to estimate the geographical distributions of TEP in the area.

At the horizontal scale, TEP was also positively related to O_2_, POC, and BP. The covariance between all these variables at the horizontal scale suggests physical forces that drive them all. We observed maxima of TEP in epipelagic waters near the coast and at the interface between slope and basin waters. In this area, which is located next to the Catalan front, a salinity doming is frequently observed (Font et al., 1988; Estrada and Salat, 1989), and relatively high Chla and PP values are found due to increased deep ocean nutrient availability at shallower depths (Estrada, 1996; Pedrós-Alió et al., 1999). This is, however, not evident in our dataset since some basin stations (i.e., stations 8 and 9) were sampled during stormy conditions that rendered more mixed temperature and salinity profiles, while the rest were sampled in sunny days and well-stratified profiles.

### TEP vertical distribution in epipelagic waters

In contrast to horizontal TEP distributions, vertical TEP distributions could not be predicted from Chla at the vertical scale. In this case, TEP maxima were always located shallower than the DCM. TEP maxima above the DCM and near the surface have been reported in previous studies in the Alboran Sea (García et al., 2002), the Eastern Mediterranean Sea (Bar-Zeev et al., 2011), and along a West to East transect in the Mediterranean Sea (Ortega-Retuerta et al., 2010). Furthermore, in a recent study (Kodama et al., 2014), TEP maxima were associated to layers of maximum O_2_ and nutrient minima, similar to what we observed in our study. Correlation analyses confirmed the Chla-TEP decoupling. From the whole set of variables, those that explained TEP distribution in both slope and basin waters were O_2_ (positively related to TEP) and nitrate (negatively related to TEP, Table 2). This suggests that TEP are a direct product of PP, which is frequently decoupled from Chla concentration and whose highest rates are located at depths shallower than the DCM (Estrada et al., 1993). This suggests that Chla is not the best proxy of phytoplankton biomass or PP at the vertical scale. Indeed, increases in Chla concentration at the DCM reflect photoacclimation to low light levels through increases in the Chla/carbon ratio, and do not necessarily match highest phytoplankton biomass (Delgado et al., 1992; Gernez et al., 2011).

Given that we lacked direct PP estimates, in this study we consider O_2_ as a proxy of PP in this area. We are aware, however, that O_2_ concentration in the ocean is a result of biological processes as well as physical processes, such as ventilation in the upper mixed layer. But a non-significant correlation between O_2_ and temperature indicates that O_2_ distribution in the area majorly reflects biological processes. Furthermore, in surface waters of our study, TEP and O_2_ were not significantly correlated, which supports our view that the O_2_-TEP correlation is through PP with O_2_ being a proxy of the latter. Similarly, the absence of negative correlations between O_2_ and bacterial abundance or production suggests that respiration by heterotrophs is not the main driver of O_2_ distributions.

The observed significant positive correlation with bacterial production would suggest one of the following mechanisms: (1) bacteria act as a source of TEP, (2) bacterial colonization and utilization of TEP, or (3) dependence of both TEP and bacteria on other factors, namely organic compounds released by phytoplankton during primary production. We performed multiple regression analyses with TEP as the dependent variable and O_2_ and BP as independent variables. Both variables significantly explained TEP variability (*r*^2^ = 0.71, *p* = 0.000, *n* = 25), with partial coefficients of 0.72 for O_2_ and 0.46 for BP, respectively. Additionally, we correlated the raw residuals of the O_2_-TEP regression against BP, resulting significant and positive (*r* = 0.38, *p* < 0.05, *n* = 25). Both analyses lead us to conclude that both PP (with O_2_ as surrogate) and bacterial activity (with BP as surrogate) has a significant influence on vertical TEP distributions. Finally, the correlation between TEP and the BP/O_2_ ratio was significant and positive. The positive correlation indicates that the higher the bacterial processing of organic matter originated in primary production, the higher TEP concentrations we observed, suggesting then a synergy between carbon fixation and bacterial reutilization of this fixed C to generate TEP.

Nutrients may also have an impact on TEP distributions as suggested by the negative correlations with NO_3_, PO_4_, and SiO_4_. TEP are enriched in carbon respect to N and P (Mari et al., 2001), and different experimental studies have demonstrated a higher TEP release rate when nutrients are limiting (Mari et al., 2005; Pedrotti et al., 2010). Also, the N/P ratios are likely important in determining TEP production and degradation, as suggested by a significant correlation between the N/P ratio and TEP concentration in epipelagic waters. Although how nutrient stoichiometry affects TEP dynamics is unclear and probably dependent on the composition of the initial microbial community (Gärdes et al., 2012); our negative correlation suggests that the lower the relative proportion of P, the higher the TEP; which contrasts with previous experimental results (Engel et al., 2015). However, the likely limitation by phosphorus in our system (e.g., Sala et al., 2002; Pinhassi et al., 2006) in contrast to the Atlantic area studied by Engel et al. (2015) could differently affect TEP production. Indeed, it has been proposed that extracellular organic carbon production is highest under P limitation (Mauriac et al., 2011). TEP properties also vary depending on whether they are released during active growth or during bloom senescence (Mari et al., 2001), with implications for the fate of these particles in seawater (degradation vs. export). In the NW Mediterranean, PP usually peaks at the end of winter and spring (Gasol et al., 2016), so we sampled during the transition of spring to summer, which coincides with the beginning of nutrient depletion and associated decreases in PP. Thus, we expected TEP to accumulate and be prone to further export.

### TEP vertical distribution in meso- and bathy-pelagic waters

In meso- and bathypelagic waters TEP distributions were only explained by turbidity changes. Specifically, we could detect TEP increases associated with near-bottom particle-rich layers (BNL). Surprisingly, these TEP increases were not paralleled by increases in POC. This suggests that these BNL were composed mostly of mineral particles that could be coated or aggregated by TEP. A previous study in the NW Mediterranean (Puig et al., 2013) observed the presence of fine particles in the BNL. Via microscope visualizations, they showed that organic matter in the BNL was mainly composed by “amorphous aggregates,” and suggested that these aggregates had lower sinking rates than phytoplankton cells or other solid organic particles. This is in line with our findings, where TEP, which are low density particles, may have a longer residence time in the BNL than the rest of POC compounds. In bathypelagic waters, TEP cannot have a direct phytoplanktonic source as light is absent. However, a bacterial source was not evident either since no significant correlations were observed between TEP and BA or BP. Interestingly, TEP concentrations in that layer were positively correlated to bacterial fucosidase activity. Since TEP are enriched in fucose (Zhou et al., 1998), this may reflect bacterial degradation of TEP in deep waters. Therefore, bacteria would act as a sink instead of a source for TEP, and a probable non-local TEP source must exist, material either sunk from epipelagic waters, resuspended from the sediment, or advected off the shelf.

### TEP diel variations

To our knowledge this is the first time that high frequency (every 4 h) and short-term (i.e., 2-day) TEP changes have been monitored in the field during a lagrangian study. However, we did not find a recurrent pattern of any of the variables measured. In our case, this lack of diel patterns of microbial biomass/activity likely explained the absence of recurrent TEP diurnal or nocturnal maxima. Additionally, although we confirmed in this study the vertical decoupling between Chla and TEP and the better coincidence with O_2_ concentrations, the results of short-term variations of these variables were less conclusive. We got some hints about short-term temporal decoupling, where TEP peaks followed Chla and O_2_ peaks, but further work, with longer diel sampling, is needed to explore this issue.

## Conclusions

We showed that the TEP-Chla relationship in the ocean is variable and mainly depends on the time and spatial scale studied. TEP can be predicted from Chla distributions at the horizontal scale, which opens the possibility to estimate surface TEP using remote sensing Chla; but this relationship is not evident at the vertical scale, nor at a short timescale, and also likely varies seasonally. Since our dataset is limited, in an attempt to compare our results to other areas, we compiled information on the various TEP-Chla relationships published in the literature (Table 3). The previous results mainly concur with our observations: TEP patterns mimic Chla patterns horizontally, but they are vertically decoupled, and TEP concentration maxima are frequently found at depths shallower than the DCM. Hence, we propose O_2_ concentrations and bacterial production as predictive variables for vertical TEP distributions in the ocean. Further, we suggest looking at the spatial and temporal variations of TEP together with primary productivity measurements and orienting further work to elucidate what is the specific role of bacterioplankton at explaining geographical and vertical TEP distributions in the ocean.

**Table 3:** **Review of TEP-Chla relationships available in the literature**.

**Scale**	**Year**	**Season**	**Area**	**Depth**	**TEP range**	**Chl range**	**Correlation**	***r***	**References**
Horizontal	1997	Spring	Gulf of Cadiz	10 m			+	0.60	Prieto et al., 2006
		Spring		25 m			+	0.33	
		Spring		Euphotic	63.8–202.4		+	0.29	
		Spring		Aphotic					
		Spring	NW Alboran Sea				+	0.92	
	2006	Spring	Bay of Biscay	0–160 m	1.41–31.7^*1^	0.8–2.1	+	0.61	Harlay et al., 2010
	2006–2008	Spring	Celtic Sea	0–80 m	22–1101	0.39-1.44	+	0.42	Van Oostende et al., 2012
	2012	Spring	NW Mediterranean Sea	0–200 m	9.9–24.9		+	0.75	This study
	1999–2002	All year	Adriatic Sea	Surface	4–14000	1–14	+		Radic et al., 2005
Vertical	2008–2009	Spring–summer	Eastern mediterranean Sea		51–290	0.02–0.99	–	−0.69	Bar-Zeev et al., 2011
	2009	Spring	North Pacific	Mixed layer	5.1–65.4	0.30–1.72	ns		Wurl et al., 2011
	2009	Summer	Hawaii	Mixed layer	<2.5–60.2	0.38–0.63	ns		
	2009	Fall	Arctic	Mixed layer	7.9–117.9	0.57–7.80	ns		
	2009-2010	Summer–winter	Pearl River Estuary		88.7–1586	na	ns		Sun et al., 2012
	2012	Spring	NW mediterranean Sea	0-2300	4.9–54.9	0–1.73	ns		This study
	2013	Spring	NW Pacific	Epipelagic	35.3–47.2	0.05–0.26	ns		Kodama et al., 2014
Hor-vert	2007	Spring	Mediterranean Sea	0–200 m	4.5–94.3	0–1.78	ns		Ortega-Retuerta et al., 2010
	2014	Spring	Mediterranean Sea	Surface-deep			ns		Mazuecos, 2015, thesis
	2005	Summer	Southern ocean	0-200 m	0–48.9	0.01–5.36	+	0.52	Ortega-Retuerta et al., 2009b
	2010–2011	All	Tropical and subtropical World oceans	Surface-deep			ns		Mazuecos, 2015, thesis
		Summer	Gibraltar Strait	0–75 m	169.3		+	0.73	Prieto et al., 2006
Temporal	1997–1998	All	Tokio Bay	0–10 m	14–1774	<5–30	+	0.65	Ramaiah and Furuya, 2002
	1998–2000	All	Dona Paula Bay	Surface	1.3–149.1	1.2–12.3	ns		Bhaskar and Bhosle, 2006
	1999–2000	All	NW Mediterranean Sea	Surface-DCM	Nd^*2^	0–2.8	+	0.71	Beauvais et al., 2003
	2012	Spring-summer	English channel	Surface and microlayer	254–1301	0.5–5.5	ns		Taylor et al., 2014
		Spring-summer	English channel	Surface	36.9–1735.1	9	+^*3^	0.87	Klein et al., 2011
	2002–2004	Summer–winter	Aegean Sea	Surface to 4 m (bottom)	101–259	0.1–7	+	0.19	Scoullos et al., 2006

*Horizontal-vertical scales (hor-vert in the Table) are considered when analyses are made using data that covered wide geographical areas but also include deep vertical profiles. ns, not significant. na, not available*.

*1 *Relative units (samples not calibrated)*.

*2 *TEP analyzed by microscopic enumeration*.

*3 *Analysis only with chl >10 μm*.

## Author contributions

EO, MS, FP, RS, CM, and JG designed the work. EO, CM, FA, CA, RG, EB, and MM sampled and performed laboratory analyses and processed the data. EO wrote the manuscript with the help and inputs of all co-authors.

### Conflict of interest statement

The authors declare that the research was conducted in the absence of any commercial or financial relationships that could be construed as a potential conflict of interest.

## Supplementary material

Supplementary Figure 1 **Bivariate plot between depth-averaged (upper mixed layer) temperature (abscises) and salinity (ordinates) in the 13 sampling stations along the diel cycle**.

Supplementary Figure 2 **Variations of dissolved inorganic nitrogen (DIN, orange circles), dissolved inorganic phosphorus (DIP, green-yellow triangles), and estimated bacterial production with factor 1.55 kgC mol leucine (BP, purple triangles) in the coastal transects in May (A)** and June **(B)**.
