# Supplementary figures and images for: Horizontal and Vertical Distributions of Transparent Exopolymer Particles (TEP) in the NW Mediterranean Sea Are Linked to Chlorophyll a and O2 Variability
Source: Front Microbiol. 2017 Jan 31;7:2159. doi: 10.3389/fmicb.2016.02159 (PMC5281620; doi:10.3389/fmicb.2016.02159)

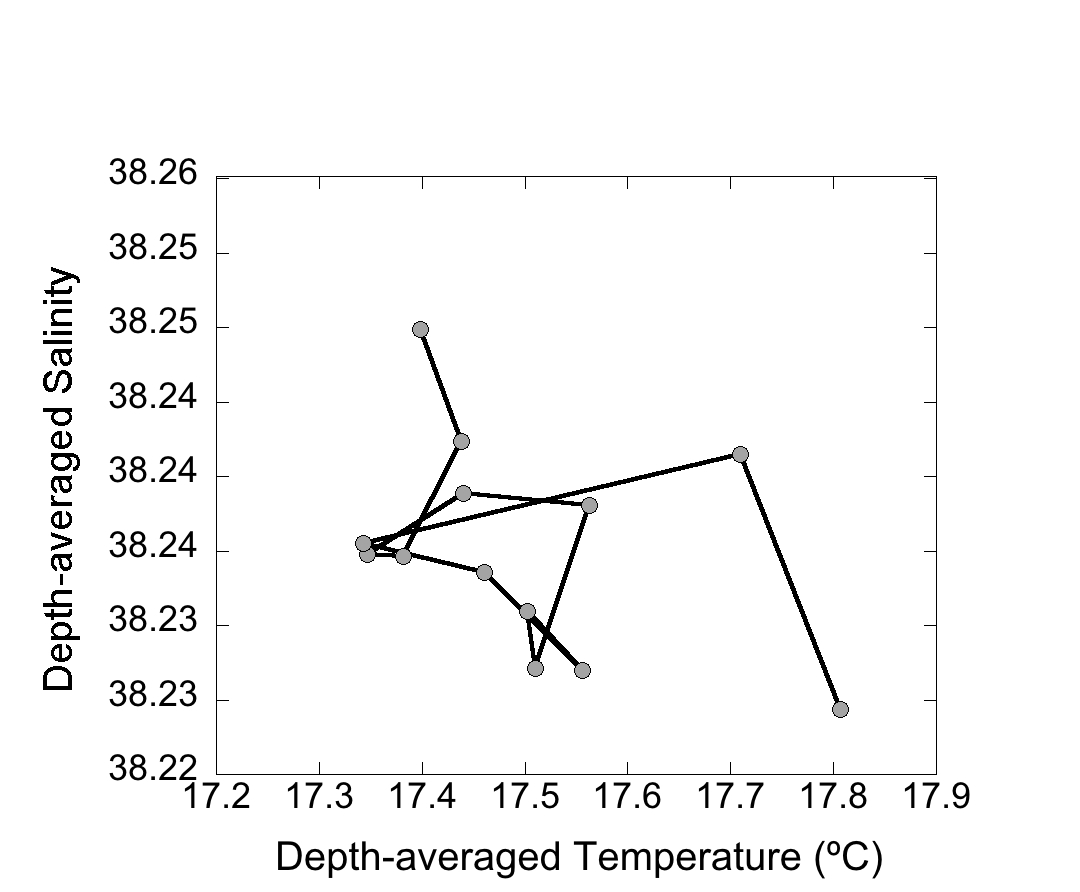

Supplement: Supplementary Figure 1 — Bivariate plot between depth-averaged (upper mixed layer) temperature (abscises) and salinity (ordinates) in the 13 sampling stations along the diel cycle. [file Image1.JPEG]

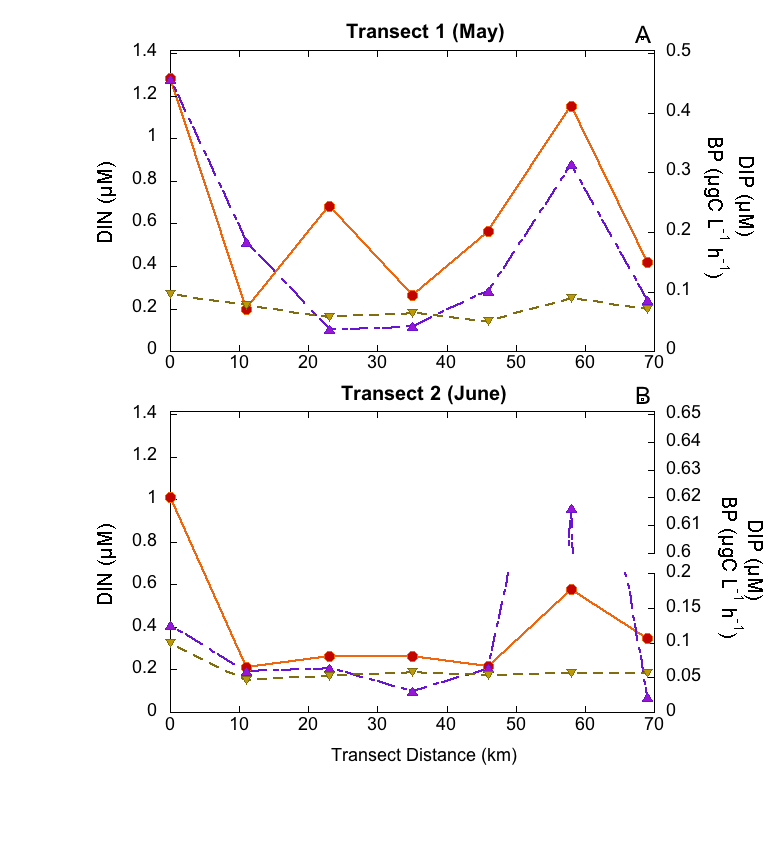

Supplement: Supplementary Figure 2 — Variations of dissolved inorganic nitrogen (DIN, orange circles), dissolved inorganic phosphorus (DIP, green-yellow triangles), and estimated bacterial production with factor 1.55 kgC mol leucine (BP, purple triangles) in the coastal transects in May (A) and June (B). [file Image2.tif]
